# A Gate-and-Switch Model for Head Orientation Behaviors in *Caenorhabditis elegans*

**DOI:** 10.1523/ENEURO.0121-18.2018

**Published:** 2018-12-10

**Authors:** Marie-Hélène Ouellette, Melanie J. Desrochers, Ioana Gheta, Ryan Ramos, Michael Hendricks

**Affiliations:** 1 Department of Biology, McGill University, Montreal, Quebec H3A 1B1, Canada

**Keywords:** behavior, *C. elegans*, calcium signaling, sensorimotor integration

## Abstract

The nervous system seamlessly integrates perception and action. This ability is essential for stable representation of and appropriate responses to the external environment. How the sensorimotor integration underlying this ability occurs at the level of individual neurons is of keen interest. In *Caenorhabditis elegans*, RIA interneurons receive input from sensory pathways and have reciprocal connections with head motor neurons. RIA simultaneously encodes both head orientation and sensory stimuli, which may allow it to integrate these two signals to detect the spatial distribution of stimuli across head sweeps and generate directional head responses. Here, we show that blocking synaptic release from RIA disrupts head orientation behaviors in response to unilaterally presented stimuli. We found that sensory encoding in RIA is gated according to head orientation. This dependence on head orientation is independent of motor encoding in RIA, suggesting a second, posture-dependent pathway upstream of RIA. This gating mechanism may allow RIA to selectively attend to stimuli that are asymmetric across head sweeps. Attractive odor removal during head bends triggers rapid head withdrawal in the opposite direction. Unlike sensory encoding, this directional response is dependent on motor inputs to and synaptic output from RIA. Together, these results suggest that RIA is part of a sensorimotor pathway that is dynamically regulated according to head orientation at two levels: the first is a gate that filters sensory representations in RIA, and the second is a switch that routes RIA synaptic output to dorsal or ventral head motor neurons.

## Significance Statement

Simple head orientation behaviors in *Caenorhabditis elegans* provide an opportunity to dissect sensorimotor integration at the cellular and subcellular levels. We describe a single interneuron that coordinates posture, sensation, and movement to detect the spatial distribution of stimuli and direct motor output to guide navigation behavior. Our findings underscore the fundamental role of neural mechanisms for integrating sensory input into ongoing behavior.

## Introduction

Perception and action are intimately linked in the central nervous system. Our movements often have immediate sensory consequences, including changes in visual, proprioceptive, and mechanosensory inputs. Likewise, vocalization produces auditory self-stimuli, and inhalation can result in olfaction. To ensure accurate internal representations of the world, reafferent sensory stimulation caused by our own behavior must be processed and interpreted differently from other stimulus sources (Straka et al., 2018). To make this distinction, our sensory systems must continuously predict the consequences of our own actions. This is in part accomplished via inputs from motor areas, called efference copies or corollary discharge (Crapse and Sommer, 2008). Through processes that are only partially understood, our senses are precisely filtered and modulated in real time to compensate for ongoing behaviors and the sensory consequences of our actions. While some of the neural circuits that underlie these functions have been anatomically characterized to varying degrees, the sites and molecular mechanisms of this type of sensorimotor integration remain elusive in all but a few cases. The nematode *Caenorhabditis elegans*, which has a completely mapped and largely invariant nervous system, quantifiable behaviors, and a suite of genetic and imaging tools, provides an opportunity to probe these events at cellular resolution and directly link them to behavior (White and Southgate, 1986; Calhoun and Murthy, 2017).

The primary mode of *C. elegans* navigation is a biased random walk consisting of bouts of forward movement punctuated by quasi-random reorientations achieved through clustered sequences of reversals and turns. Directional movement is achieved by measuring changes in sensory input over time during forward bouts and modulating reorientation probability accordingly (Pierce-Shimomura et al., 1999). This behavior is directly analogous to the runs and tumbles of bacterial chemotaxis (Berg, 1975). *C. elegans* lie on their side, undulating in the dorsoventral plane as they crawl in a sinusoidal motion. Sensory structures located at the tip of the nose sweep back and forth as the head bends during forward movement. In contrast to the biased random walk, several navigation behaviors suggest that reafferent sensory input caused by head bending during locomotion is exploited as a way to sample the spatial distribution of environmental stimuli and guide navigation, a simple form of active sensing. For example, in radial temperature gradients, *C. elegans* will crawl in precisely curved trajectories to stay within a favored temperature range, a behavior called isothermal tracking (Hedgecock and Russell, 1975). Likewise, when crawling in a chemoattractant gradient, *C. elegans* exhibit gradual steering to orient in the preferred direction, a behavior called klinotaxis, as long as they are at an angle relative to the gradient that allows sensory sampling across head sweeps (Iino and Yoshida, 2009; Kato et al., 2014). Steering and the biased random walk operate on fundamentally different principles (Lockery, 2011). While the latter operates primarily by sensory integration over tens of seconds (Pierce-Shimomura et al., 1999; Luo et al., 2014a,b), steering requires constant, ongoing integration of sensory and motor information on a short time scale (Iino and Yoshida, 2009; Kato et al., 2014).

**Figure 1.**
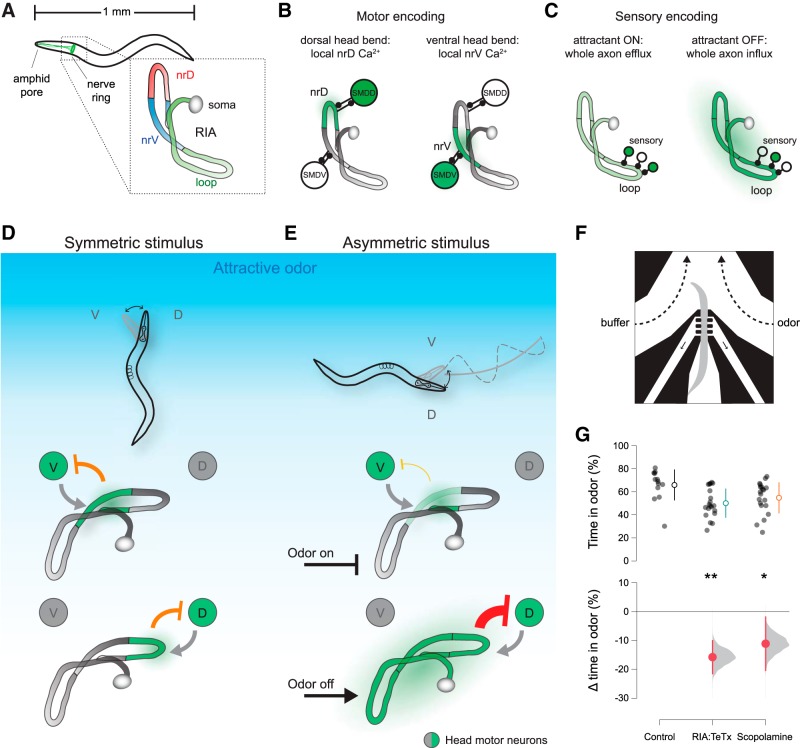
RIA overview and requirement for control of head orientation. ***A***, RIA is a unipolar interneuron whose axon extends into the ventral nerve cord and throughout the nerve ring. There are two RIAs, left and right, one is shown. ***B***, Two axonal compartments within the nerve ring, nrV and nrD, encode head movements via local calcium signals (mCa^2+^) triggered by muscarinic input from SMD head motor neurons. ***C***, Sensory pathways synapse on the loop domain of the RIA axon in the ventral nerve cord. Attractive stimuli lead to whole-axon reduction in calcium, while attractant removal causes whole axon calcium increases (sCa^2+^). ***D***, Model for RIA function in steering behaviors. Where stimuli are symmetric across head bends, inhibitory output from RIA to motor neurons is symmetric. ***E***, If the stimulus changes across head sweeps, RIA output to motor neurons become asymmetric, driving steering behavior. ***F***, Schematic of head orientation measurement in response to a unilaterally presented stimulus. ***G***, Estimation plot of difference between wild-type preference for odor as measured by percentage of the assay time (120 s) with their head within the odor stream and RIA::TeTx animals or animals treated with scopolamine. ANOVA *F*_(2,50)_ = 5.7771, *p* = 0.0055, *p < 0.05, **p < 0.01 *post hoc* Student’s *t* test, *n* = 13, *n* = 20, *n* = 20. V, ventral; D, Dorsal. Open circles and lines are mean ± SD (top). Closed circles and lines are mean difference from control ± 95% confidence interval, shown with distribution of estimated means (see Materials and Methods).

We previously showed that each of a pair of unipolar interneurons called RIA can simultaneously encode both head movements and sensory inputs through two types of calcium signal (Hendricks et al., 2012). Local, compartmentalized calcium events in the ventral and dorsal segments of the RIA axon (nrV and nrD) were correlated with dorsal and ventral head bends, respectively (Fig. 1*A*,*B*). These axonal compartments correspond to sites of synaptic input from two pairs of head motor neurons, the SMDs (White and Southgate, 1986). We will refer to these compartmentalized events within the nerve ring as mCa^2+^, for motor-evoked calcium events. In addition to mCa^2+^ signals, removal of an attractive odor resulted in calcium increase throughout the entire axon, including nrV and nrD, while odor presentation resulted in whole-axon suppression of calcium levels (Fig. 1*C*). These events will be referred to as sCa^2+^, for sensory-evoked calcium events. These responses are dependent on synaptic input from upstream sensory neurons and interneurons that synapse onto RIA exclusively in the “loop” region of the axon in the ventral nerve cord (Fig. 1*A*).

Within the nerve ring, mCa^2+^ and sCa^2+^ signals overlap spatially and temporally. We previously showed that these signals were genetically separable, and they were additive with one another within each nerve ring compartment. This additivity suggests a simple mechanism for integrating head orientation and sensory input to calculate the spatial distribution of a stimulus across each head sweep. We therefore proposed a model for steering based on asymmetric output from RIA to head motor neurons (Hendricks and Zhang, 2013). The basis of this model is that RIA mediates negative feedback onto head motor neurons, limiting the extent of head bending and setting gait amplitude. This is supported by the observation that impairing RIA function led to increased gait amplitude (Hendricks et al., 2012). When stimuli are absent or distributed symmetrically (Fig. 1*D*), this negative feedback is also symmetric. However, if stimulus changes are experienced across head bends, as when crawling orthogonal to a gradient, sCa^2+^ events are produced in phase with head bends, leading to enhanced negative feedback in the unfavored direction and suppressed negative feedback in the favored direction (Fig. 1*E*).

Here, we confirm and extend aspects of this model by examining simple head orientation behaviors and sensory encoding events in RIA, which have not been explored in detail and which reveal new features of sensorimotor integration in RIA. We propose a “gate and switch” model for RIA function in which sensory filtering prevents inappropriate activation of RIA when the head is not bent (gating), while mCa^2+^ asymmetry constitutes a switch that routes an inhibitory signal to dorsal or ventral motor neurons to bias gait. Finally, we identify explanatory shortcomings of the model that require further study.

## Materials and Methods

### Animals

*C. elegans* were raised on nematode growth medium and fed with *Escherichia coli* strain OP50 according to standard methods (Brenner, 1974; Stiernagle, 2006). Experiments were conducted on young (3 d old) adult hermaphrodites. The strains used are N2, VC657 *gar-3(gk305)*V, MMH099 *yxIs19 [Pglr-3a::GCaMP3.3, Punc-122::DsRed]*, MMH100 *yxIs19; yxIs20 [Pglr-3::TeTx:mCherry, Punc-122::GFP]*; VC657 *gar-3(gk305)*V; ZC1792 *gar-3(gk305)*V; ZC1923 *gar-3(gk305)V; yxIs19; yxEx968 [Pglr-3::gar-3]*. Molecular biology and strain generation was previously described (Hendricks et al., 2012). *Pglr-3::TeTx:mCherry* was generated by LR recombination of *pSM-rfB-TeTx:mCherry* destination and *pCR8-Pglr-3* entry Gateway vectors described therein (Invitrogen), followed by injection at 25 ng/μl. Tetanus toxin is expressed as a C-terminal fusion protein with mCherry. MMH099 is derived from the previously described strain ZC1508 after extensive outcrossing to N2. Some strains were provided by the *Caenorhabditis* Genetics Center (CGC).

### Microfluidic device fabrication

Standard soft lithography methods were used to fabricate photoresist (SU8) masters for microfluidic devices (San-Miguel and Lu, 2013). Two-component polydimethylsiloxane (PDMS; Dow Corning Sylgard 184, Ellsworth Adhesives #4019862) was mixed at 10:1 w/w, degassed under vacuum, poured over masters, degassed again, and cured at 60°C for at least 3 h. Devices were replica mastered in a two-part epoxy resin (Smooth Cast 310, Sculpture Supply Canada #796220) according to the manufacturer's instructions. Inlet holes were made with a Milltex 1 mm biopsy punch (Fisher). Chips were cleaned with ethanol and Scotch tape (3M) and then bonded to glass coverslips using air plasma generated by a handheld corona treater (Haubert et al., 2006; Electro-Technic Products). Coupling to fluid reservoirs was done by directly inserting PTFE microbore tubing (Cole-Parmer #EW-06417-21) into inlet holes.

### Calcium imaging

Fluorescence time lapse imaging (100-ms exposures, five frames per second) was performed as described (Hendricks et al., 2012). Briefly, animals were restrained in a microfluidic channel with a depth of 28 μm to prevent movement in the *z*-axis and that allows the anterior portion of the head to move freely (Chronis et al., 2007). They were exposed to alternating streams of NGM buffer (1 mM CaCl_2_, 1mM MgSO_4_, and 25 mM KPO_4_; pH 6.0) or 100 μM isoamyl alcohol (IAA; BioShop ISO900) in NGM buffer. Fluid flow was driven by negative pressure from a vacuum pump and controlled with a ValveBank (AutoMate Scientific). Images were captured at five frames per second, 100-ms exposures using a 40× objective. Movements in xy were corrected by stack registration, and ROIs corresponding to the RIA axonal domains were selected manually. GCaMP3 (Tian et al., 2009) intensity was measured from axonal compartments and normalized with the formula (F_t_ – F_min_)/(F_max_ – F_min_). Asymmetry between the nerve ring compartments (∂nr) was defined as (nrV – nrD). All calcium imaging analysis was done using the FIJI distribution of ImageJ (Schindelin et al., 2012). During calcium imaging, animals were free to move the anterior portion of their head, and these movements were captured as well. Motion artifacts affecting the GCaMP3 signal can in principle produce spurious correlations with the animal’s movement. If our correlations were artifacts of animal movement, these correlations should persist in *gar-3* mutants or in scopolamine-treated animals, in which there are no compartmentalized mCa^2+^ events. However, both in previous work using an identical imaging configuration (Hendricks, 2012) and here, correlations between head movement and local GCaMP3 signals are completely eliminated, demonstrating that they are not produced by motion artifacts.

### Head orientation assays

Microfluidic chips (60 μm deep) were based on a design by McCormick et al. (2011). Fluid flow was gravity driven and controlled with manual one-way stopcocks (Cole-Parmer). Animals were exposed to streams of NGM buffer and 100 μM IAA on either side of their heads. For scopolamine treatment, animals were immersed in NGM buffer containing 100 μM scopolamine (Sigma) for 5 min before and during the assay. Worms were loaded individually into the chip, and once in place, their movements were recorded for 2 min, with a switch between buffer and IAA at the 1-min mark, using a USB camera (Aven Mighty Scope, #2700-200) and VirtualDub software.

### Head orientation measurements

In head orientation assays, head bending was measured at each time point as the angle between the tip of the animal’s nose and the midline of its body at the most anterior restrained point using MATLAB. For calcium imaging, head bending angle was calculated as the angle between the major axis of an ellipse fit to the free portion of the animal’s head and neutral (unbent) position using FIJI. Comparison to previously used methods based on the ratio of pixels on either side of the midline (Hendricks et al., 2012) showed that these approaches yield equivalent results.

### Code accessibility

MATLAB code used to measure head orientation in Figure 1 is available from the authors on request.

### Statistics and data presentation

All time series data are presented as lines with shading indicating the standard error of the mean. Where comparisons are made, we have adopted the use of estimation plots, which focus on effect sizes and confidence intervals (Gardner and Altman, 1986; Ho et al., 2018). Estimation plots employ a secondary axis that shows the size of the difference between test condition and control as a mean and 95% confidence interval. For paired comparisons, this is the within-group difference. A probability distribution of the test mean was produced by generating 2500 Bayesian bootstrapped means using sampling with replacement from the experimental data (Rubin, 1981). The implementation used here weighted each observed mean according to a gamma distribution with shape parameter (*n* – 1)/*n* and a scale parameter of 1. Where multiple comparisons are made, significance markers are for *post hoc* Student’s *t* test with Bonferroni correction. All statistical analysis and plotting were done in JMP Pro 13 (SAS). Figures were prepared with Adobe Illustrator.

## Results

### RIA and head orientation

Ablating RIA does not grossly affect random walk dynamics in isotropic environments or in response to olfactory stimuli (Gray et al., 2005; Ha et al., 2010). In contrast to the temporal integration of the biased random walk, steering behavior depends on asymmetric head bends that propagate through the normal locomotor body wave, producing curved forward movement (Iino and Yoshida, 2009; Izquierdo and Lockery, 2010; Lockery, 2011). To test the idea that RIA is involved in biasing head movements in response to an asymmetrically presented olfactory stimulus, we analyzed head movements in a microfluidic chip designed for this purpose (Fig. 1*F*). In this device, animals are restrained in approximately the middle third of their body using gentle suction. Two fluid streams flow on either side of the head. Animals exhibit spontaneous head bends and body undulations and are able to express a preference for one of the two streams by restricting their movements to keep their head in the preferred odor stream (McCormick et al., 2011).

Because our previous characterization of RIA physiology used the chemoattractant IAA (3-methylbutan-1-ol) and steering has been demonstrated in IAA gradients, we used it as an olfactory stimulus (Hendricks et al., 2012; Kato et al., 2014; Liu et al., 2018). We recorded each animal for two minutes. To control for any intrinsic bias in head bending direction, the odor and buffer streams were switched after one minute. Wild-type animals showed a clear preference for the IAA stream, as measured by the proportion of time spent with their heads bent toward the odor (Fig. 1*G*).

To test the role of RIA in this behavior, we used animals expressing tetanus toxin under an RIA-specific promoter. Tetanus toxin prevents synaptic vesicle release by cleaving synaptobrevin (Schiavo et al., 1992). In contrast to wild type, RIA::TeTx animals exhibited no preference (Fig. 1*G*). Next, we treated animals with scopolamine, a muscarinic acetylcholine receptor antagonist previously shown to abolish mCa^2+^ events (Hendricks et al., 2012). Like RIA::TeTx animals, scopolamine treated animals show no directional preference (Fig. 1*G*). RIA-defective animals exhibit normal sCa^2+^ responses and RIA-ablated animals are capable of olfactory chemotaxis and thus are not sensory impaired (Ha et al., 2010; Hendricks et al., 2012). These results are consistent with the hypothesis that RIA is involved in responses to asymmetrically presented stimuli but not the temporal integration that underlies the biased random walk strategy.

### RIA mediates directional head withdrawal

Steering requires posture-dependent responses to sensory inputs. We therefore examined the position and velocity of the head immediately before and after IAA removal in animals restrained in a chip that allows the head to move while facilitating temporal control of odor presentation and calcium imaging (Chronis et al., 2007; Hendricks et al., 2012). While analyzing head movements in restrained animals prevented us from measuring navigation behavior directly, it has the advantage of allowing us to unambiguously assign a stimulus change to a specific point in the head bending cycle.

**Figure 2.**
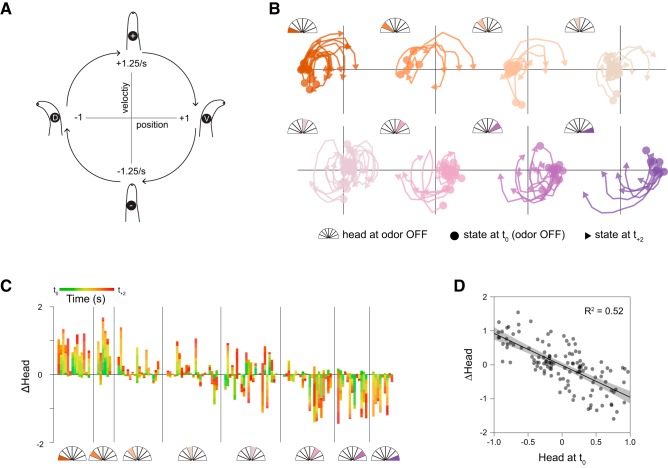
Responses to odor removal depend on posture. ***A***, Schematic of position-velocity plots to illustrate oscillatory head movements. ***B***, Position-velocity trajectories from odor removal (circles) to 2 s after (arrowheads). ***C***, Head displacement in the 2 s immediately following odor removal, with the start position normalized to head orientation at the odor switch. ***D***, Relationship between head position at odor removal and the change in head position 2 s later. Linear regression *F*_(1,124)_ = 132.3145, *p* < 0.0001; *n* = 126.

We first examined whether there was any relationship between head movements and olfactory stimulus changes. To better visualize oscillatory head bending, we plotted head movements on a 2D position-velocity space that captures its phasic properties (Fig. 2*A*). We then analyzed head movements that occurred immediately after odor removal in relation to each phase of the head bending cycle (Fig. 2*B*). Large ventral head movements were associated with odor removal during dorsal head bends, and odor removal during ventral head bends triggered rapid dorsal head movements (Fig. 2*C*).

**Figure 3.**
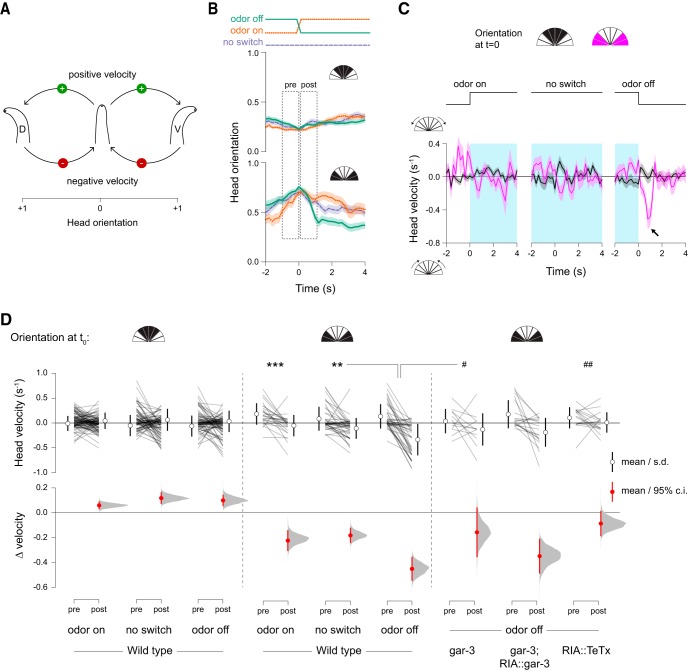
RIA mediates directional head withdrawal. ***A***, To analyze head withdrawal behaviors, which are mirror-symmetric across the dorsoventral axis, head deflection in either the dorsal (D) or ventral (V) direction is defined as positive. Positive velocities correspond to bending away from the body axis and negative velocities indicate head withdrawal. ***B***, Mean plots of peristimulus head orientation at odor off, odor on, or in constant odor (no switch), binned according to whether the head is unbent (top) or bent in either direction (bottom) at the time of stimulus change (*t* = 0). Dashed “pre” and “post” boxes indicate time windows used for quantitation in ***D***. ***C***, Comparison of head velocity in response to stimulus changes (or no switch) when the head is bent or unbent. Arrow indicates characteristic head withdrawal in response to odor removal when the head is bent. ***D***, Paired mean pre- and post-switch head velocities (upper panels, open circles and lines are mean ± SD) and estimations of the size of the head velocity change (lower panel, closed circles and lines are mean difference ± 95% confidence interval, along with probability distribution of means, see Materials and Methods). Odor switch (on, off, or constant odor) has no effect on head movements when the head is not bent (left panel, repeated measures ANOVA *F*_(2,263)_ = 0.9386, *p* = 0.3925, *n* = 100, *n* = 82, *n* = 84). Sharp decreases in velocity are seen when odor removal occurs when the head is bent, but not for odor presentation or constant odor (middle panel, repeated measures ANOVA, *F*_(2,109)_ = 7.7932, *p* = 0.0007, *n* = 26, *n* = 44, *n* = 42). In *gar-3* mutants and animals expressing tetanus toxin in RIA these sharp decreases in head velocity are absent (right panel, repeated measures ANOVA *F*_(3,85)_ = 4.4105, *p* = 0.0062, *n* = 42, *n* = 12, *n* = 18, *n* = 17); ***p < 0.001, **p < 0.01 *post hoc* Student’s *t* test between odor conditions (on, off, no switch) for wild-type animals; ##*p* < 0.01, #*p* < 0.05 *post hoc* Student’s *t* test between genetic manipulation (wild type, *gar-3*, *gar-3* rescue, RIA::TeTx) for odor off responses during head bending.

Because locomotion involves regular, oscillatory head movements, head withdrawal (returning toward center) is expected to follow immediately after each head bend. We predicted that stimulus-evoked movements would alter this normal oscillation. To distinguish the latter active head withdrawal events from normal behavioral progression through the oscillatory cycle, we compared head movements before and after odor withdrawal to head movements associated with either odor presentation or at an arbitrary time point with no stimulus change. Because responses to stimuli should be mirrored across the dorsoventral axis, for these experiments we defined head deflection in either direction as positive. Thus, positive velocities correspond to head bending and negative velocities to head withdrawal (Fig. 3*A*). While head bending does tend to be followed by head withdrawal (Fig. 3*B*), as expected, only odor removal elicits a characteristic, transient, rapid head movement and only when the head is bent (Fig. 3*C*,*D*).mCa^2+^ events rely on synaptic input from SMD motor neurons via the muscarinic AChR (mAChR) GAR- 3 (Hendricks et al., 2012). Our model suggests that these events play a role in modulating head movements in response to sensory stimuli. We therefore examined stimulus-evoked head withdrawal in *gar-3* mutants and in RIA::TeTx animals. Neither of these strains exhibited stimulus-evoked head withdrawals (Fig. 3*D*). Expressing a wild-type *gar-3* cDNA specifically in RIA showed partial rescue of odor removal-evoked head withdrawal, but these responses were not significantly different from those of *gar-3* mutants (Fig. 3*D*).

### RIA sensory responses are gated according to head position

Models of steering rely on symmetry breaking by phasic modulation of the effects of sensory input on motor networks (Lockery, 2011). Support for a role for RIA in this process was provided in a recent paper in which selective inactivation of RIA synapses in either nrV or nrD produced curved locomotion in the predicted direction (Liu et al., 2018). This relies on generating deeper head bends in the direction of curvature, essentially the inverse of head withdrawal. Both mechanisms rest on the idea that differential mCa^2+^ levels in nrV and nrD function as a switch to route output from RIA to ventral or dorsal motor neurons. This output is predicted to be inhibitory and to limit head bends in the unfavorable direction. However, restricting head bends must be balanced against the need to maintain forward locomotion. Because mCa^2+^ levels are only partially correlated with head movements and frequently do not show dorsal-ventral differences (Hendricks et al., 2012), there is a potential for inappropriate symmetrical activation or inhibition, which would be predicted to disrupt the animal’s gait.

**Figure 4.**
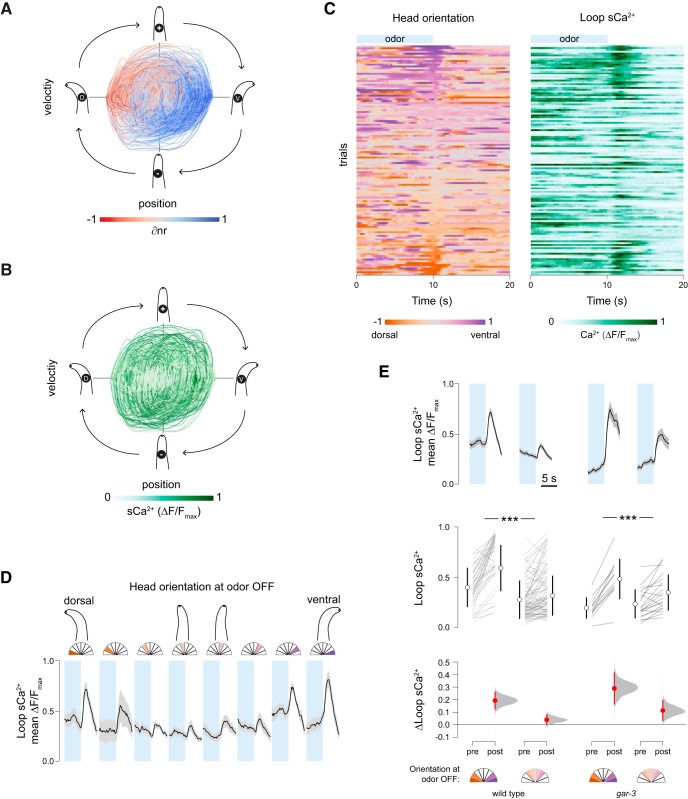
Head orientation gates sensory responses. ***A***, Head position-velocity trajectories color-coded by ∂nr (= nrV – nrD), a measure of normalized mCa^2+^ asymmetry in nrV and nrD. ***B***, Head position-velocity trajectories color-coded by loop calcium signal, *n* = 126, 40 s each. ***C***, Spontaneous head movements (left) and sCa^2+^ signals in the loop region of the RIA axon (right). Rows are matched and sorted according to head orientation at odor off (*t* = 10 s). A linear regression of loop response magnitudes and head deflection in either direction was significant (*F*_(1,124)_ = 29.98, *p* < 0.0001, *n* = 126). ***D***, Mean traces of loop calcium responses 5 s before and after odor removal binned by head orientation at the odor off time point. Shading is SEM. Left to right, *n* = 14, *n* = 6, *n* = 18, *n* = 22, *n* = 23, *n* = 21, *n* = 13, *n* = 9. ***E***, Comparison of the magnitude of loop calcium responses to odor removal when the head is bent (head orientation > 0.5 or < –0.5) or unbent (head orientation between –0.5 and 0.5). Pre- and post-measurements are mean loop Ca^2+^ levels in 1s windows just before odor OFF and centered on the peak of the mean response, respectively, shown as paired responses (lines) and mean ± SD (open circles and lines). Estimations of the mean changes are shown below as mean difference ± 95% confidence interval (closed circles and lines) along with probability distributions of the means (see Materials and Methods). In both wild-type (*n* = 84, *n* = 42) and *gar-3* (*n* = 20, *n* = 12) animals, the loop response magnitude is larger when the head is bent (repeated measures ANOVA *F*_(1,154)_ = 41.4529, *p* < 0.0001, ***p < 0.001 *post hoc* Student’s *t* test). *gar-3* mutants have larger responses under both head orientation conditions, but there is no significant interaction between loop Ca^2+^ responses, head position at odor off, and genotype (repeated measures ANOVA *F*_(1,154)_ = 0.2145, *p* = 0.6439).

We therefore analyzed the relationship between head movements, mCa^2+^ asymmetry in the nerve ring, and sCa^2+^ responses in the loop domain. Consistent with previous observations, mCa^2+^ asymmetry (∂nr) shows a clear relationship to head oscillations (Fig. 4*A*). There is no apparent overall relationship between loop sCa^2+^ and head movement (Fig. 4*B*). Because odor switches occur at set time points while head movements are spontaneous, the relationship between head position and odor removal is arbitrary. Spontaneous sCa^2+^ events may mask a relationship between head movement and stimulus-evoked events because the latter are much less common. We therefore examined whether stimulus-evoked loop sCa^2+^ events that occur on odor removal are dependent on head phase.

We found an unexpected relationship between loop responses and head position at the time of odor removal. Odor removal triggers calcium influx throughout the RIA axon via inputs from upstream interneurons, and the magnitude of these responses was large when the animal’s head was bent, in either direction, and small or absent when it was not (Fig. 4*C*,*D*). This effect is the same for dorsal or ventral bends, so as in Figure 3, we defined all bends as positive and classified head positions as “bent” or “not bent” if they were more or less than half the maximal degree of head deflection, respectively. This posture classification revealed a robust difference in the magnitude of sCa^2+^ responses in response to odor removal (Fig. 4*E*).

Posture dependence in behavioral responses to pulsed thermal stimuli was observed by Stephens et al. (2008). In that study, animal posture was defined through a dimensional reduction procedure, and an animal’s state defined by the first two components was predictive of responses to a sudden temperature increase. With only a behavioral readout, it is difficult to tell whether posture dependence is based on an active neural mechanism or is a constraint of the motor program. In the case of RIA, we have clear evidence of a sensory gating mechanism. This has important implications for sensory coding in RIA. First, gating allows preferential attention to stimuli encountered during head bends, consistent with a function in steering in response to asymmetrically distributed stimuli. Second, it resolves the issue raised above of potential gait disruption caused by symmetrical output from RIA to head motor neurons. Gating ensures that large sCa^2+^ events are only likely to occur when mCa^2+^ in the nerve ring is asymmetric, i.e., only when the downstream “switch” is engaged to favor either dorsal or ventral synaptic output.

The sensory gating mechanism may act directly on RIA or be a feature of upstream interneurons. We first tested the hypothesis that the gate and switch mechanisms are in fact the same: motor inputs via GAR-3 that produce local mCa^2+^ events may also somehow gate sCa^2+^ responses. To do so we repeated our analysis of sensory-evoked sCa^2+^ relative to head orientation in *gar-3* mutants. In this mutant, mCa^2+^ events are absent while sCa^2+^ responses are intact, leading to perfectly symmetrical calcium activity in the nerve ring (Hendricks et al., 2012). *gar-3* mutants exhibited normal sensory gating (Fig. 4*E*). This suggests that an independent gating mechanism exists at the level of the RIA loop or upstream interneurons. There are several candidate mechanoreceptor neurons in the head, and this mechanism may rely on a hitherto unidentified proprioceptive pathway.

### Head bending desynchronizes local calcium responses to sensory input

How sCa^2+^ and mCa^2+^ interact and influence synaptic release is critical for understanding of RIA function. We previously showed that sCa^2+^ and mCa^2+^ are additive within the nerve ring compartments (Hendricks et al., 2012). Therefore, we analyzed nrV and nrD responses separately during whole-axon sCa^2+^ events with respect to each other and head velocity.

**Figure 5.**
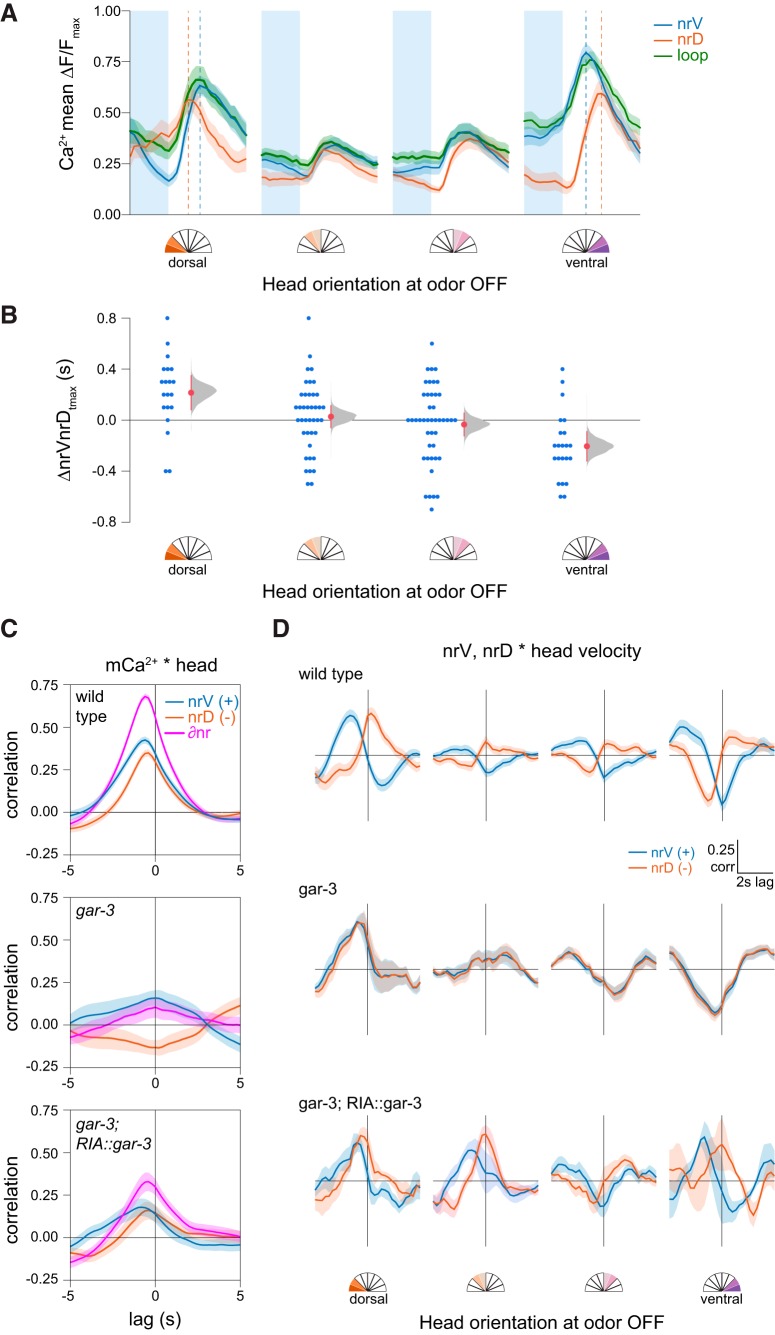
Temporal features of sensory responses in RIA axonal compartments. ***A***, Mean nrV, nrD, and loop responses on odor removal. Shading is SEM, dashed lines indicate position of nrV and nrD mean peaks. ***B***, Head orientation predicts the mean lag between nrV and nrD peaks post-odor removal. ANOVA *F*_(1,124)_ = 13.47, *p* = 0.0004. ***C***, Cross-correlation of nrV, nrD, and mCa^2+^ asymmetry (∂nr) and head orientation, showing mCa^2+^ lag with head movements. *gar-3* mutants lack mCa^2+^ and do not show head correlations. Re-expression of *gar-3* cDNA in RIA partially rescues this relationship. ***D***, nrV and nrD cross-correlations with head velocity for wild-type, *gar-3*, and RIA-specific *gar-3* rescue in a 6-s time window comprising 2-s pre-odor removal and 4-s post-odor removal in relation to head orientation at odor off. When the head was bent, we observed no lag between head withdrawal and peak calcium responses in the nerve ring compartment ipsilateral to the direction of bending. *gar-3* mutants show no distinction between nerve ring compartments. Expression of *gar-3* cDNA in RIA in *gar-3* mutants does not rescue the temporal features of sensory responses in the nerve ring. Analysis groups are the same as Figure 4.

We found that head bending introduced a notable lag in peak responses within the nerve ring that was dependent on head orientation (Fig. 5*A*,*B*). The polarity of this lag depended on head position, such that the ipsilateral nerve ring compartment exhibited a shorter rise time and earlier peak with respect to the contralateral compartment. This is consistent with a simple additive model of mCa^2+^ and sCa^2+^. That is, the nerve ring compartment corresponding to the direction of head bending exhibits not only a stronger calcium signal but a shorter latency to peak calcium levels.

As previously shown, we observed that mCa^2+^ responses typically lag behind head movements (Fig. 5*C*). Possible reasons for this lag include the relative timing of the muscle contractions (via nicotinic acetylcholine receptors) and mCa^2+^ (via muscarinic receptors) elicited by acetylcholine released from head motor neurons. We have interpreted this lag as evidence that mCa^2+^ in RIA function as a representation of ongoing head movement. To understand how the shift in peak local calcium (the sum of sCa^2+^ and mCa^2+^ calcium sources within each compartment) might relate to stimulus-evoked head withdrawal, we examined the relative timing of these events. We performed cross-correlation analysis of head velocities with respect to nrV and nrD calcium dynamics in a restricted time window corresponding to odor removal. In these plots, positive peaks near the *y*-axis indicate a calcium signal that is coincident with head motion. Those to the left are calcium signals that are delayed relative to head motion, and thus are unlikely to play a causal role in the initiation of the behavior.

During sensory stimulation (odor removal) while the head is bent, peak sensory responses in the nerve ring are coincident with head withdrawal in the contralateral direction but lagged in the ipsilateral direction (Fig. 5*D*). That is, when the head is bent ventrally, the peak nrV calcium signal is synchronous with the highest head velocity in the dorsal direction (withdrawal) while nrD calcium lags, and vice versa during dorsal head bends. When the head is not bent, this relationship is lost due to the absence of strong sCa^2+^ responses. This desynchronization is absent in *gar-3* mutants, which completely lack mCa^2+^ (Hendricks et al., 2012). Re-expression of *gar-3* specifically in RIA restored some compartmentalization (Fig. 5*D*) but was not sufficient to rescue the temporal structure of stimulus-evoked responses in nrV and nrD, which may relate to its failure to fully rescue head withdrawal (Fig. 4*D*). This implies either GAR-3 plays additional roles outside RIA in mediating the temporal features of RIA signaling and head withdrawal, or that the rescue construct causes mislocalization, inappropriate expression levels, or otherwise does not sufficiently restore GAR-3 function in RIA.

Overall, these results show that the additive properties of mCa^2+^ and sCa^2+^ lead to decoupling of local calcium event timing in the nerve ring in response to odor removal. This has important functional implications for mCa^2+^. Because these signals slightly lag head movement, it was unclear how they might play an instructive role in controlling rapid response to sensory stimulus. Our results suggest that one possible role is to function additively with sCa^2+^ to reduce latency and facilitate more rapid transmitter release on one side of the RIA axon than the other, triggering head withdrawal.

## Discussion

RIA is uniquely well-situated in the *C. elegans* wiring diagram to play a role in integrating head orientation and sensory input. It is one of very few interneurons that receive substantial motor feedback, and it receives convergent input from odor, taste, and temperature sensory pathways. Here, we confirmed a role for RIA in head orientation in response to an asymmetrically presented stimulus, following previous work demonstrating roles for RIA in steering in response to odor and temperature (Mori and Ohshima, 1995; Liu et al., 2018).

The recent study by Liu et al. (2018) demonstrated that asymmetrically suppressing neurotransmitter release from RIA, mimicking an attractive odor being presented on that side, led to deeper head bends and curving in that direction. Here, we describe the mirror-image behavior, head withdrawal and limiting of head bends in response to odor removal. Both are predicted by our model to contribute to steering (Fig. 1). Blocking RIA synaptic release or genetically removing asymmetric local mCa^2+^ in the RIA axon prevented directional head withdrawal. We identified a posture-dependent sensory gating mechanism that may prevent aberrant symmetric RIA output and causes RIA to be selectively responsive to stimuli encountered during head bending.

Phasic behaviors that produce reafferent stimulation often exhibit sensory gating coupled to the behavior. In mice, examples include olfaction, where cholinergic inputs to the olfactory bulb modulate sensory gain in phase with respiration during sniffing, and a functionally similar gating mechanism is evident during whisking (Eggermann et al., 2014; Rothermel et al., 2014). RIA may be part of a similar circuit mechanism, in which phasic inputs from head motor networks both gate sensory responses and tune motor outputs to respond appropriately to spatially asymmetric stimuli.

**Figure 6.**
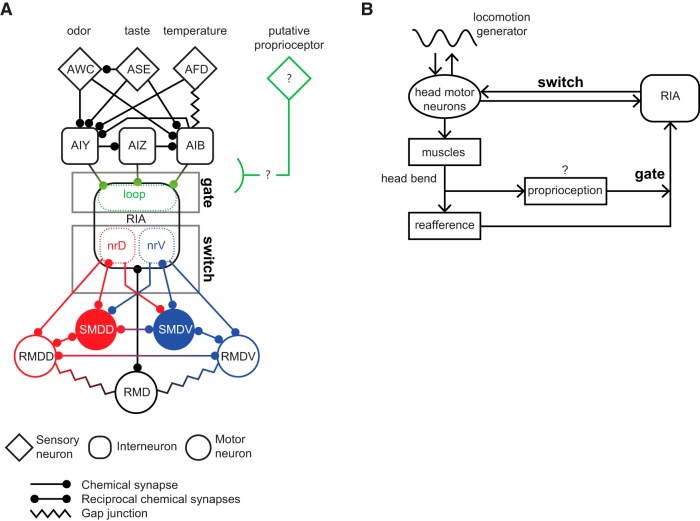
Gate and switch model for RIA function in head orientation behaviors. ***A***, RIA circuit diagram, with major upstream sensory pathways and head motor connections. Gating of sensory inputs to the loop domain and the nerve ring connections that constitute the dorsal-ventral switch. ***B***, Ethological model in which head movements simultaneously produce proprioception-gated reafference and efference copies that converge on RIA. RIA output to motor neurons biases the undulatory gait dorsally or ventrally when reafferent stimuli are asymmetric across head sweeps.

A shortcoming of our model is that the rapid head withdrawal movements we observed, which often involve sudden reversals of head velocity, probably cannot be explained by inhibition of ipsilateral motor neurons alone but must also involve contralateral motor neuron activation and muscle contraction. In the nerve ring, the RIA axon receives input from ventral head motor neurons only in nrV and dorsal motor neurons only in nrD. However, output synapses from RIA to SMD and RMD head motor neurons are not segregated in the same way. nrV and nrD both contain interspersed output synapses to both dorsal and ventral motor neurons. However, the postsynaptic anatomic arrangement of these synapses suggest that these connections may be of opposite valence (Fig. 6*A*). Ipsilateral connections are in the distal portion of the axon near neuromuscular junctions, while contralateral synapses are clustered near the motor neuron cell body. This is at least consistent with the possibility of differing effects of output from nrV and nrD on their ipsilateral contralateral motor neuron targets. Exploration of this will require identification of postsynaptic receptors and direct measurement of RIA’s effects on its target neurons.

Our observation of dynamic sensory gating adds a new layer to the RIA sensorimotor circuit and resolves potential shortcomings of our previous model, in which RIA outputs but not sensory representations are regulated by head movements. We propose a two-level gate and switch model, in which an unidentified posture-dependent signal gates sensory sCa^2+^ responses while differential mCa^2+^ between the nerve ring compartments functions as a switch to produce asymmetric synaptic output from the nrV and nrD axonal domains (Fig. 6*A*,*B*). This switch may depend on the temporal decoupling we observed between nrV and nrD during sensory-evoked responses when the head is bent. mCa^2+^ introduces a temporal bias that reduces the time to peak response in the compartment contralateral to the direction of head withdrawal. This peak is coincident with head withdrawal, and the duration of the behavior is similar to the lag introduced between the two axonal compartments. Again, how plausible a mechanism this is will depend on analyzing the effects of these calcium events on synaptic release from RIA and the activity of postsynaptic motor neurons.

Despite its small size and simple morphology, RIA exhibits striking computational potential as a sensorimotor integrator. Along with clear behavioral readouts, this relative simplicity makes it an attractive system to probe sensorimotor integration at the cellular level, both in terms of molecular mechanisms and circuit logic. This work underscores the fundamental role of circuits that link self-motion and ongoing behavior to interpretation of the sensory environment.

## Synthesis

Reviewing Editor: Miriam Goodman, Stanford University Decisions are customarily a result of the Reviewing Editor and the peer reviewers coming together and discussing their recommendations until a consensus is reached. When revisions are invited, a fact-based synthesis statement explaining their decision and outlining what is needed to prepare a revision will be listed below. The following reviewer(s) agreed to reveal their identity: Mei Zhen.

This is an interesting manuscript that investigates the neural mechanisms that integrate external sensory stimuli and re-afferent sensory information. It continues the functional characterization of the RIA interneuron, initially reported by the Hendricks et al. Nature 2012, with a recent follow-up by Liu et al., Neuron 2018. RIA is a set of interneurons in the C. elegans head that exhibits complex calcium dynamics such that their single neurites exhibit compartmentalized calcium signals linked both sensory and motor information.

In this study, the authors first confirm RIA's in driving steering in response to olfactory or thermosensory gradients, re-examining spatial-temporal dynamics in RIA calcium signaling with the same sensory stimuli, genetically-encoded calcium sensor, and the same microfluidics. The results demonstrate that sensory stimulation affects RIA calcium levels only when the head is in a bent position. They show that RIA gates olfactory information based on head position, such that odorant-evoked calcium responses are suppressed when the head is not bent. They further show that removal of an attractive odorant causes rapid head withdrawal, and that this response requires motor input into RIA and also output from RIA. The investigation of RIA's role in isothermal tracking, a motor program that requires fine temporal and spatial integration by the sensorimotor system that is essential for efficient thermotaxis, is a key advance. Experiments support earlier reports that RIA plays a key role in thermotaxis, demonstrating that loss of RIA signaling impairs isothermal tracking by reducing run length.

The review team agreed that is a really nice manuscript, but that it would be very difficult for readers new to the behaviors and neurons under study to digest. In discussion, the reviewers agreed that a revised manuscript would need to include a more accessible digest of the prior work, the model for RIA function and contribution to gating sensory stimulation based upon gait. For example, the authors write (line 74) ”A model was proposed for steering based on asymmetric output from RIA to head motor neurons (Hendricks and Zhang, 2013)“ in the introduction, but do not describe the model for readers. For the discussion, please endeavor to compare this study to prior work emphasizing points of agreement as well divergence and novelty. The discussion focuses primarily on calcium imaging results, but would benefit from a broader synthesis including the implications of the effect of disrupting RIA synaptic output on isothermal tracking.

Additionally, the explanations of methods and results provided in the manuscript are very terse.

It was also noted that the study fell short on revealing the circuit or molecular mechanisms by which RIA executes the motor gating. However, they excluded an essential involvement of GAR-3-dependent acetylcholine signaling or reciprocal wiring with the head motor neurons by RIA. These negative results are informative, leading these authors to speculate on the involvement of unknown proprioceptors.

Both reviewers supported the publication of this study, and provided additional specific comments to assist the authors with their revision of the text.

1. Overall this manuscript was densely written. There was insufficient introduction of the background and context of this work, as well as insufficient description of materials, methods, and results. This would make this manuscript very difficult to follow by anyone that does not have intimate knowledge of the system already.

2. Starting with an essential role of RIA in isothermal tracking was disjoint from the rest of the study. Is quantification of the forward run length alone a sufficient analysis of the isothermal tracking phenotype of RIA-defective worms? A reduced ability to sustain a long forward run alone is probably not the most informative metric of isothermal tracking performance. Shouldn't there be other correlated parameters such as the head angle during steering? Along the same line, authors stated that RIA-TeTx animals did not exhibit motor defects in isotropic environments, but the toxicity of overexpressing TeTx in other neurons can lead to broad motor defects such as reduced run length. Authors should have quantified data that support this statement.

3. Additional details are needed in Materials and Method details regarding these points:

o How many animals were analyzed in each isothermal tracking assay?

o How were the different transgenic strains constructed? For example, was the RIA::TeTx::mCherry construct an RIA::TeTx::SL2::mCherry construct or was the TeTx tagged? More detail about each of the constructs generated for this paper would be very helpful.

o How were movement artefacts accounted for during calcium imaging, especially since ratiometric imaging was not performed? Was movement constrained enough along the z-axis that movement artefacts were not an issue?

o For the radial gradient assay, how accurately is the temperature gradient maintained for the 20 min assay duration?

4. And, the following display items need additional explanation

o Figure 1D: It would be helpful to see normal movement of RIA::TeTx worms in an isothermal environment. Also, the wild-type image is a bit confusing. Is that a second worm on the bottom of the plate? The movement of that worm doesn't look different from that of the RIA::TeTx worm.

o Figure 2A: It's unclear which side the odor is coming from, and how that relates to head movement.

o Figure 2D: Why is the head orientation below 0.5? Does this mean the worm has a preferred head position?

o Figure 2E: It's confusing the way velocity in the two directions is being binned together. The y-axis is no longer showing velocity but is labeled as velocity, which is confusing.

o Figure 2F-H: What time point is being shown? The time point at which the velocity is being measured should be clearly stated.

o Figure 3C: The time axis isn't labeled.

o Figure 4C and D: More explanation of these figures, and what exactly is being quantified, is necessary.

5. There are a number of grammatical and spelling mistakes throughout. Please be vigilant in correcting these errors in the revision.

6. Additional specific comments from one of the reviewers can be found in the pdf file attached to this review.

## References

[B1] Berg HC (1975) Bacterial behaviour. Nature 254:389–392. 1090851 10.1038/254389a0

[B2] Brenner S (1974) The genetic of *Caenorhabditis elegans* . Genetics 77:71–94. 4366476 10.1093/genetics/77.1.71 PMC1213120

[B3] Calhoun AJ, Murthy M (2017) Quantifying behavior to solve sensorimotor transformations: advances from worms and flies. Curr Opin Neurobiol 46:90–98. 10.1016/j.conb.2017.08.006 28850885 PMC5765764

[B4] Chronis N, Zimmer M, Bargmann CI (2007) Microfluidics for in vivo imaging of neuronal and behavioral activity in *Caenorhabditis elegans* . Nat Methods 4:727–731. 10.1038/nmeth1075 17704783

[B5] Crapse TB, Sommer MA (2008) Corollary discharge across the animal kingdom. Nat Rev Neurosci 9:587–600. 10.1038/nrn2457 18641666 PMC5153363

[B6] Eggermann E, Kremer Y, Crochet S, Petersen CCH (2014) Cholinergic signals in mouse barrel cortex during active whisker sensing. Cell Rep 9:1654–1660. 10.1016/j.celrep.2014.11.005 25482555

[B7] Gardner MJ, Altman DG (1986) Confidence intervals rather than P values: estimation rather than hypothesis testing. Br Med J 292:746–750. 3082422 10.1136/bmj.292.6522.746 PMC1339793

[B8] Gray JM, Hill JJ, Bargmann CI (2005) A circuit for navigation in *Caenorhabditis elegans* . Proc Natl Acad Sci USA 102:3184–3191. 10.1073/pnas.0409009101 15689400 PMC546636

[B9] Ha HI, Hendricks M, Shen Y, Gabel CV, Fang-Yen C, Qin Y, Colón-Ramos D, Shen K, Samuel ADT, Zhang Y (2010) Functional organization of a neural network for aversive olfactory learning in *Caenorhabditis elegans* . Neuron 68:1173–1186. 10.1016/j.neuron.2010.11.025 21172617 PMC3038580

[B10] Haubert K, Drier T, Beebe D (2006) PDMS bonding by means of a portable, low-cost corona system. Lab Chip 6:1548–1549. 10.1039/b610567j 17203160

[B11] Hedgecock EM, Russell RL (1975) Normal and mutant thermotaxis in the nematode *Caenorhabditis elegans* . Proc Natl Acad Sci USA 72:4061–4065. 1060088 10.1073/pnas.72.10.4061 PMC433138

[B12] Hendricks M, Ha H, Maffey N, Zhang Y (2012) Compartmentalized calcium dynamics in a *C. elegans* interneuron encode head movement. Nature 487:99–103. 10.1038/nature11081 22722842 PMC3393794

[B13] Hendricks M, Zhang Y (2013) Complex RIA calcium dynamics and its function in navigational behavior. Worm 2:e25546. 10.4161/worm.25546 24778936 PMC3875648

[B36] Ho J, Tumkaya T, Aryal S, Choi H, Claridge-Chang A (2018) Moving beyond P values: Everyday data analysis with estimation plots. bioRxiv. Advance online publication. Retrieved from July 26, 2018. doi: 10.1101/377978. 10.1038/s41592-019-0470-3 31217592

[B14] Iino Y, Yoshida K (2009) Parallel use of two behavioral mechanisms for chemotaxis in *Caenorhabditis elegans* . J Neurosci 29:5370–5380. 10.1523/JNEUROSCI.3633-08.2009 19403805 PMC6665864

[B15] Izquierdo EJ, Lockery SR (2010) Evolution and analysis of minimal neural circuits for klinotaxis in *Caenorhabditis elegans* . J Neurosci 30:12908–12917. 10.1523/JNEUROSCI.2606-10.2010 20881110 PMC3422662

[B16] Kato S, Xu Y, Cho CE, Abbott LF, Bargmann CI (2014) Temporal responses of *C. elegans* chemosensory neurons are preserved in behavioral dynamics. Neuron 81:616–628. 10.1016/j.neuron.2013.11.020 24440227 PMC4112952

[B17] Liu H, Yang W, Wu T, Duan F, Soucy E, Jin X, Zhang Y (2018) Cholinergic sensorimotor integration regulates olfactory steering. Neuron 97:390–405.e3. 10.1016/j.neuron.2017.12.003 29290549 PMC5773357

[B18] Lockery SR (2011) The computational worm: spatial orientation and its neuronal basis in *C. elegans* . Curr Opin Neurobiol 21:782–790. 10.1016/j.conb.2011.06.009 21764577 PMC3947813

[B20] Luo L, Cook N, Venkatachalam V, Martinez-Velazquez LA, Zhang X, Calvo AC, Hawk J, MacInnis BL, Frank M, Ng JHR, Klein M, Gershow M, Hammarlund M, Goodman MB, Colón-Ramos DA, Zhang Y, Samuel ADT (2014a) Bidirectional thermotaxis in *Caenorhabditis elegans* is mediated by distinct sensorimotor strategies driven by the AFD thermosensory neurons. Proc Natl Acad Sci USA 111:2776–2781. 10.1073/pnas.1315205111 24550307 PMC3932917

[B21] Luo L, Wen Q, Ren J, Hendricks M, Gershow M, Qin Y, Greenwood J, Soucy ER, Klein M, Smith-Parker HK, Calvo AC, Colón-Ramos DA, Samuel ADT, Zhang Y (2014b) Dynamic encoding of perception, memory, and movement in a *C. elegans* chemotaxis circuit. Neuron 82:1115–1128. 10.1016/j.neuron.2014.05.010 24908490 PMC4082684

[B22] McCormick KE, Gaertner BE, Sottile M, Phillips PC, Lockery SR (2011) Microfluidic devices for analysis of spatial orientation behaviors in semi-restrained *Caenorhabditis elegans* . PLoS One 6:e25710. 10.1371/journal.pone.0025710 22022437 PMC3192130

[B23] Mori I, Ohshima Y (1995) Neural regulation of thermotaxis in *Caenorhabditis elegans* . Nature 376:344–348. 10.1038/376344a0 7630402

[B24] Pierce-Shimomura JT, Morse TM, Lockery SR (1999) The fundamental role of pirouettes in *Caenorhabditis elegans* chemotaxis. J Neurosci 19:9557–9569. 10531458 10.1523/JNEUROSCI.19-21-09557.1999 PMC6782915

[B25] Rothermel M, Carey RM, Puche A, Shipley MT, Wachowiak M (2014) Cholinergic inputs from Basal forebrain add an excitatory bias to odor coding in the olfactory bulb. J Neurosci 34:4654–4664. 10.1523/JNEUROSCI.5026-13.2014 24672011 PMC3965788

[B27] San-Miguel A, Lu H (2013) Microfluidics as a tool for *C. elegans* research. WormBook. Advance online publication. Retrieved from September 24, 2013. doi: 10.1895/wormbook.1.162.1. 10.1895/wormbook.1.162.1 PMC4781173 24065448

[B29] Schiavo G, Benfenati F, Poulain B, Rossetto O, Polverino De Laureto P, DasGupta BR, Montecucco C (1992) Tetanus and botulinum-B neurotoxins block neurotransmitter release by proteolytic cleavage of synaptobrevin. Nature 359:832–835. 10.1038/359832a0 1331807

[B30] Schindelin J, Arganda-Carreras I, Frise E, Kaynig V, Longair M, Pietzsch T, Preibisch S, Rueden C, Saalfeld S, Schmid B, Tinevez J-Y, White DJ, Hartenstein V, Eliceiri K, Tomancak P, Cardona A (2012) Fiji: an open-source platform for biological-image analysis. Nat Methods 9:676–682. 10.1038/nmeth.2019 22743772 PMC3855844

[B31] Stephens GJ, Johnson-Kerner B, Bialek W, Ryu WS (2008) Dimensionality and dynamics in the behavior of *C. elegans* . PLoS Comput Biol 4:e1000028. 10.1371/journal.pcbi.1000028 18389066 PMC2276863

[B32] Stiernagle T (2006) Maintenance of *C. elegans* . WormBook. Advance online publication. Retrieved from February 11, 2006. doi: 10.1895/wormbook.1.101.1. 10.1895/wormbook.1.101.1 PMC4781397 18050451

[B33] Straka H, Simmers J, Chagnaud BP (2018) A new perspective on predictive motor signaling. Curr Biol 28:R232–R243. 10.1016/j.cub.2018.01.033 29510116

[B34] Tian L, Hires SA, Mao T, Huber D, Chiappe ME, Chalasani SH, Petreanu L, Akerboom J, McKinney SA, Schreiter ER, Bargmann CI, Jayaraman V, Svoboda K, Looger LL (2009) Imaging neural activity in worms, flies and mice with improved GCaMP calcium indicators. Nat Methods 6:875–881. 10.1038/nmeth.1398 19898485 PMC2858873

[B35] White JG, Southgate E (1986) The structure of the nervous system of the nematode *Caenorhabditis elegans* . Philos Trans R Soc Lond B Biol Sci 314:1–340. 22462104 10.1098/rstb.1986.0056

